# Memory-type variance estimators using exponentially weighted moving average statistic in presence of measurement error for time-scaled surveys

**DOI:** 10.1371/journal.pone.0277697

**Published:** 2023-11-09

**Authors:** Muhammad Nouman Qureshi, Osama Abdulaziz Alamri, Naureen Riaz, Ayesha Iftikhar, Muhammad Umair Tariq, Muhammad Hanif

**Affiliations:** 1 School of Statistics, University of Minnesota, Minneapolis, Minnesota, United States of America; 2 Statistics Department, Faculty of Science, University of Tabuk, Tabuk, Saudi Arabia; 3 Department of Mathematics, Lahore Garrison University, Lahore, Pakistan; 4 Lahore Business School, University of Lahore, Lahore, Pakistan; 5 Department of Statistics, National College of Business Administration and Economics, Lahore, Pakistan; The University of Dodoma, UNITED REPUBLIC OF TANZANIA

## Abstract

The present study suggested memory-type ratio and product estimators for variance estimation in the presence of measurement errors. We applied the exponentially weighted moving averages statistic which simultaneously utilizes the current and prior information for better estimation in surveys based on the time-scale. The expressions of approximate mean square errors of memory-type estimators are derived using Taylor series up to first order. Mathematical conditions are also obtained for which the suggested memory-type ratio and product estimators perform better than the conventional ratio and product estimators. The efficiency of the proposed estimators is observed using an extensive simulation study in the presence of measurement errors. A real data application is also carried out to support the mathematical expressions. From the results, it is shown that the use of prior sample information significantly increased the efficiency of the proposed estimators.

## 1. Introduction

Surveys are relatively inexpensive and useful to describe the characteristics of large populations. It plays a vital role in many disciplines to gather the information for the estimation of unknown parameters of the population. Many surveys have been designed and administered in many modes and repeated under the similar approach with equal time space all over the globe. For instance, the Australian Bureau of Statistics and the National Bureau of Statistics of China conducted health, economic and agricultural surveys annually, quarterly, and monthly. Every year, the Pakistan Bureau of Statistics conducts the Labor Force Survey (LFS) to measure the different statistics of Pakistan. Bureau of Statistics, Punjab, has been conducting Multiple Indicator Cluster Surveys (MICS) regularly since 2004.

In survey sampling, statisticians usually effort to enhance the performance of the estimators by using the auxiliary information correlated with the study variable. In practice, the researchers disposed to get information on many variables rather than the study variable only. Ratio-type estimators are considered to be good example for the situation having a positive correlation between the auxiliary variable and the study variable whereas product-type estimators are assumed to be better for the negatively correlated variables.

The estimation of variance is important when the variability control is difficult in its application such as in agricultural research, experiments in biosciences, manufacturing industries, and pharmaceutical laboratories. The estimation of population variance in the study variable also received significant interest from various authors such as [1–11].

Measurement errors (*MEs*) are defined as the difference between observed observations and the true observations of the study variable [12]. It has severe effects on the estimation of population parameters in terms of increased bias and variation. It is essential to study the impact of *ME* to develop better estimation techniques and to get more reliable and efficient estimates of the underlying population parameters. Many authors have studied the effect of *ME* along with the estimation of population parameters. One may refer to [13–18]. Several researchers like [19–24] contribute to the variance estimation of the concerned variable in the presence of *ME*.

Initially, [25] introduced the idea of *EWMA* statistic and recycled the current and previous information to observe the change in the process mean. The memory-type ratio and product estimators for mean estimation were suggested by [26] using *EWMA* statistic under simple random sampling without replacement (*SRSWOR*). Similarly, [27] proposed the memory type ratio and product estimators for population mean using stratified sampling.

The *EWMA* statistic for population variance estimation of the study variable with 0≤*λ*≤1 is defined as:

$$V_{t}=\lambda {\hat{s}}_{yt}^{2}+(1-\lambda )V_{t-1},$$

where ${\hat{s}}_{yt}^{2}$ is the current sample variance and *λ* is the weighted parameter or smoothing constant of the given observations. As *λ* increases from 0 to 1, the larger weight is given to current information and simultaneously smaller weight to the past observations. For *λ* = 1, all the weight is received by current information and *EWMA* statistic performs the same as the conventional variance estimator. Here, *t* represents the sample numbers and *V*_*t*−1_ reveals the past observations. The initial value *V*_0_ is taken as one or the average value of the prior sample.

In this study, memory-type ratio and product estimators are suggested in the presence of *ME* for the time-scaled surveys. After a brief discussion on surveys, auxiliary information, variance estimation, *ME*, and *EWMA* statistics, the rest of the paper is as follows. Section 2 is based on the sampling scheme, notations, and conventional estimators under *ME*. Proposed estimators along with the derivation of approximate mean square error (*MSE*) are given in Section 3. Mathematical comparisons between the proposed memory-type estimators and the corresponding conventional estimators are given in Section 4. A simulation study is conducted in Section 5 to assess the performance of the proposed estimators over the conventional estimators. Real data application is also used in Section 6 to validate the findings of the mentioned estimators obtained through simulations. Final remarks are given in Section 7.

## 2. Sampling procedure and conventional estimators in the presence of *ME*

Let *Y* and *X* be the study variable and the auxiliary variable respectively defined on *N* identifiable but distinct units of a population *U* = {*U*_1_,*U*_2_,*U*_3_…,*U*_*N*_}. Let *n* pairs of observations be obtained by using *SRSWOR* on two variables *Y* and *X*. Consider a situation where both *Y* and *X* variables are observed with some considerable error. Let for the *i*^*th*^ sampling unit (*i*=1, 2 …*n*), *y*_*i*_ and *x*_*i*_ be the observed instead of true observations *Y*_*i*_ and *X*_*i*_. The *MEs* may be defined as

$$u_{i}=(y_{i}-Y_{i}).$$

and

$$v_{i}=(x_{i}-X_{i}),$$

where *u*_*i*_ and *v*_*i*_ are the associated *MEs* with constant or zero mean and known variances $S_{U}^{2}$ and $S_{V}^{2}$ respectively. Following [28], it is assumed that the errors *u*_*i*_ and *v*_*i*_ are independent of each other and also independent of *Y*_*i*_ and *X*_*i*_. This implies that *COV*(*X*, *Y*) ≠ 0 and *COV*(*X*, *U*) = *COV*(*X*, *V*) = *COV*(*U*, *Y*) = *COV*(*V*, *Y*) = *COV*(*U*, *V*) = 0. It is further assumed that the finite population correction is neglected. The expected values of $s_{y}^{2}$ and $s_{x}^{2}$ in the presence of *MEs* are defined as

$$E(s_{y}^{2})=S_{y}^{2}+S_{u}^{2}.$$

and

$$E(s_{x}^{2})=S_{x}^{2}+S_{v}^{2}.$$

It is assumed that the error variances, $S_{U}^{2}$ ad $S_{V}^{2}$ of the study and the auxiliary variables are known. The unbiased estimator of $S_{y}^{2}$ and $S_{x}^{2}$ are respectively given by

$${\hat{s}}_{y}^{2}=s_{y}^{2}-S_{u}^{2}>0.$$

and

$${\hat{s}}_{x}^{2}=s_{x}^{2}-S_{v}^{2}>0.$$

Let the sampling errors for the study and auxiliary variables are defined as

$${\hat{s}}_{y}^{2}=S_{y}^{2}(1+\xi_{o}).$$

$${\hat{s}}_{x}^{2}=S_{x}^{2}(1+\xi_{1}).$$

Such that: *E*(*ξ*_*o*_) = 0, *E*(*ξ*_1_) = 0, $E(\xi_{o}^{2})=\frac{A_{y}}{n},\;E(\xi_{1}^{2})=\frac{A_{x}}{n}$ and $E(\xi_{o}\xi_{1})=\frac{\delta -1}{n}$, where $A_{y}=\gamma_{2y}+\gamma_{2u}\frac{S_{u}^{4}}{S_{y}^{4}}+2{\left(1+\frac{S_{u}^{2}}{S_{y}^{2}}\right)}^{2},A_{x}=\gamma_{2x}+\gamma_{2v}\frac{S_{v}^{4}}{S_{x}^{4}}+2{\left(1+\frac{S_{v}^{2}}{S_{x}^{2}}\right)}^{2},\gamma_{2z}=\beta_{2z}-3$,

β2z=μ4zμ2z2,μrz=E(zi−μz)r,θx=Sx2Sx2−Sv2andδ=μ22(X,Y)Sx2Sy2,

where *z = Y*, *X*, *U*, *V*.

The derivations of the above error terms are given in the S1 Appendix.

The usual sample variance estimator with the equation of its variance in the presence of *ME* is

$$t_{y}={\hat{s}}_{y}^{2}=\frac{1}{\left(n-1\right)}\sum_{i=1}^{n}{\left(y-\bar{y}\right)}^{2}.$$

and

$$MSE(t_{r})=A_{y}\frac{S_{y}^{4}}{n}.$$

The classical ratio estimator given by [29] under *ME* is defined as

$$t_{r}=(\frac{S_{x}^{2}}{{\hat{s}}_{x}^{2}}){\hat{s}}_{y}^{2}.$$

The expression *MSE* for ratio estimator under *ME* is

$$MSE(t_{r})\approx \frac{1}{n}(A_{y}+A_{x}-2(\delta -1))S_{y}^{4},$$

Similarly, the product estimator along with the expression of *MSE* in the presence of *ME* is given as

$$t_{p}={\left(\frac{S_{x}^{2}}{{\hat{s}}_{x}^{2}}\right)}^{-1}{\hat{s}}_{y}^{2}.$$

and

$$MSE(t_{p})\approx \frac{1}{n}(A_{y}+A_{x}+2(\delta -1))S_{y}^{4}.$$

## 3. Proposed memory-type estimators

In this section, we processed the *EWMA* statistic for both study and the auxiliary variables to propose the memory-type estimators in the presence of *ME*. The *EMWA* statistic for the study variable is defined as

$${\hat{V}}_{t}=\lambda {\hat{s}}_{yt}^{2}+(1-\lambda ){\hat{V}}_{t-1}.$$

Similarly, the EWMA statistic for the auxiliary variable *x* is defined as

$${\hat{W}}_{t}=\lambda {\hat{s}}_{xt}^{2}+(1-\lambda ){\hat{W}}_{t-1}.$$

A pilot study must have reliable results before conducting every survey. The initial values of ${\hat{V}}_{t}$ and ${\hat{W}}_{t}$ are taken as average values estimated from the pilot study or taken as one. The memory-type ratio and product estimators based on *EWMA* statistic using *ME* are defined as

$${\hat{t}}_{rt}^{M}=(\frac{S_{x}^{2}}{{\hat{W}}_{t}}){\hat{V}}_{t}.$$

$${\hat{t}}_{pt}^{M}={\left(\frac{S_{x}^{2}}{{\hat{W}}_{t}}\right)}^{-1}{\hat{V}}_{t},$$

where $S_{x}^{2}$ is assumed to be the known population variance of the auxiliary variable. To derive the approximate mean square errors of both memory-type estimators, let us define the following error terms as

$$\begin{array}{c} \xi_{0t}=\frac{{\hat{V}}_{t}}{S_{y}^{2}}-1\;\mathrm{and}\;\xi_{1t}=\frac{{\hat{W}}_{t}}{S_{x}^{2}}-1\\ E(\xi_{0t})=E(\xi_{1t})=0\\ E(\xi_{0t}^{2})=\frac{Var({\hat{V}}_{t})}{S_{y}^{4}}=\frac{A_{y}}{n}(\frac{\lambda }{2-\lambda })\\ E(\xi_{1t}^{2})=\frac{Var({\hat{W}}_{t})}{S_{x}^{4}}=\frac{A_{x}}{n}(\frac{\lambda }{2-\lambda })\\ E(\xi_{0t}\xi_{1t})=\frac{\mathrm{cov}({\hat{V}}_{t},{\hat{W}}_{t})}{S_{y}^{2}S_{x}^{2}}=\frac{\left(\delta -1\right)}{n}(\frac{\lambda }{2-\lambda }) \end{array}|$$ (1)

We may rewrite the memory-type ratio estimator in terms of sampling errors as

$${\hat{t}}_{rt}^{M}={\hat{V}}_{t}(\frac{S_{x}^{2}}{{\hat{W}}_{t}})=\frac{S_{x}^{2}}{(1+\xi_{1t})S_{x}^{2}}(1+\xi_{0t})S_{y}^{2}.$$ (2)

Simplifying and applying Taylor series up to the second-order on Eq (2), we have

$${\hat{t}}_{rt}^{M}\approx (1-\xi_{1t}+\xi_{1t}{}^{2})(1+\xi_{0t})S_{y}^{2}.$$ (3)

Shortening and applying expectation on both sides of Eq (3), we have the *MSE* of ${\hat{t}}_{rt}^{M}$ as

$$MSE({\hat{t}}_{rt}^{M})\approx [\frac{Var({\hat{V}}_{t})}{S_{y}^{4}}+\frac{Var({\hat{W}}_{t})}{S_{x}^{4}}-2\frac{cov({\hat{V}}_{t},{\hat{W}}_{t})}{S_{y}^{2}S_{x}^{2}}]S_{y}^{4}.$$ (4)

The $MSE({\hat{t}}_{rt}^{M})$ in the presence of *ME* as

$$MSE({\hat{t}}_{rt}^{M})\approx (\frac{\lambda }{n(2-\lambda )})[A_{y}+A_{x}-2(\delta -1)]S_{y}^{4}.$$ (5)

Similarly, the *MSE* of the memory-type product estimator is

$$MSE({\hat{t}}_{pt}^{M})\approx [\frac{Var({\hat{V}}_{t})}{S_{y}^{4}}+\frac{Var({\hat{W}}_{t})}{S_{x}^{4}}+2\frac{\mathrm{cov}({\hat{V}}_{t},{\hat{W}}_{t})}{S_{y}^{2}S_{x}^{2}}]S_{y}^{4}.$$ (6)

The $MSE({\hat{t}}_{pt}^{M})$ in the presence of *ME* as

$$MSE({\hat{t}}_{pt}^{M})\approx (\frac{\lambda }{n(2-\lambda )})[A_{y}+A_{x}+2(\delta -1)]S_{y}^{4}.$$ (7)

## 4. Mathematical comparisons

The efficiency comparisons of the proposed memory-type estimators over the conventional estimators in terms of *MSE* are

- The proposed memory-type ratio estimator will perform better than the conventional ratio estimator if
$$MSE({\hat{t}}_{rt}^{M})<MSE(t_{r})$$
$$(\frac{\lambda }{n(2-\lambda )})[A_{y}+A_{x}-2(\delta -1)]S_{y}^{4}<\frac{1}{n}[A_{y}+A_{x}-2(\delta -1)]S_{y}^{4}.$$
$$(\frac{\lambda }{2-\lambda })<1.$$
λ<1.
- The proposed memory-type product estimator will perform better than the conventional product estimator if
$$MSE({\hat{t}}_{pt}^{M})<MSE(t_{p})$$
$$(\frac{\lambda }{n(2-\lambda )})[A_{y}+A_{x}+2(\delta -1)]S_{y}^{4}<\frac{1}{n}[A_{y}+A_{x}+2(\delta -1)]S_{y}^{4}.$$
$$(\frac{\lambda }{2-\lambda })<1.$$
λ<1.

As *λ* value ranges from 0 to 1. Therefore, under the condition, *λ*<1, the proposed memory-type estimator would always be more efficient than the conventional ratio estimator in presence of *ME*. But if we take *λ* = 1, the performance of both memory-type and usual estimators will be equal.

## 5. Simulation study

An extensive simulation study is conducted to evaluate the performance of the proposed memory-type estimators in the presence of *ME*. The *MSE* and relative efficiency (*RE*) of the proposed and existing estimators are computed by using the following formulas with 50,000 replications as

$$MSE(t_{i})=\frac{1}{50000}\overset{50000}{\underset{1}{\sum }}{\left(t_{i}-S_{y}^{2}\right)}^{2}.$$

and

$$RE(t_{i},s_{y}^{2})=\frac{Var(s_{y}^{2})}{MSE(t_{i})},$$

where $t_{i}=s_{y}^{2},t_{r},t_{rt}^{M},\;\mathrm{and}\;t_{pt}^{M}.$

The results of memory-type ratio and product estimators are computed at different levels of correlation *ρ*_*YX*_(= 0.80,0.85,0.90 and 0.95) and weight parameter *λ*(= 0.10,0.20,0.50,0.75) by using the following algorithm:

1. A population of size *N* = 5000 is generated using multivariate normal distribution having $\mu_{YXUV}=[5\;6\;0\;0],\;S_{y}^{2}=125,\;S_{x}^{2}=100,\;S_{U}^{2}=S_{V}^{2}=1\;\mathrm{and}\;2.$
2. Different values have opted for weight constant *λ*.
3. Sample of size *n* (= 50, 100, 200, 300, and 500) is considered to estimate the unknown parameter.
4. Fifty thousand iterations are used to compute the proposed estimator under *ME* from the samples obtained in Step 3.
5. The *MSE* and *RE* of each estimator are calculated for each sample size and presented in Tables 1–4.

**Table 1:** The *MSEs* of ratio-type estimators with and without *ME* for $S_{U}^{2}=1,S_{V}^{2}=1$.

with *ME*										without *ME*							
ρ_YX_	*n*	*λ* = 0.1		*λ* = 0.2		*λ* = 0.5		*λ* = 0.75		*λ* = 0.1		*λ* = 0.2		*λ* = 0.5		*λ* = 0.75	
		*t*_*r*_	$t_{rt}^{M}$	*t*_*r*_	$t_{rt}^{M}$	*t*_*r*_	$t_{rt}^{M}$	*t*_*r*_	$t_{rt}^{M}$	*t*_*r*_	$t_{rt}^{M}$	*t*_*r*_	$t_{rt}^{M}$	*t*_*r*_	$t_{rt}^{M}$	*t*_*r*_	$t_{rt}^{M}$
**0.80**	**50**	515.303	25.129	515.303	55.545	515.303	161.847	515.303	298.459	502.057	24.555	502.057	54.344	502.057	158.110	502.057	291.212
	**100**	267.557	13.386	267.557	24.880	267.557	76.953	267.557	132.623	261.195	13.087	261.195	24.309	261.195	75.252	261.195	129.596
	**200**	110.516	5.684	110.516	12.033	110.516	36.717	110.516	71.996	108.016	5.558	108.016	11.767	108.016	35.873	108.016	70.325
	**300**	74.849	3.928	74.849	7.562	74.849	25.007	74.849	41.636	73.186	3.842	73.186	7.397	73.186	24.456	73.186	40.779
	**500**	43.581	2.312	43.581	4.655	43.581	13.494	43.581	26.740	42.624	2.261	42.624	4.546	42.624	13.207	42.624	26.156
**0.85**	**50**	370.248	18.320	370.248	38.922	370.248	121.049	370.248	220.191	361.048	17.912	361.048	38.026	361.048	118.293	361.048	214.998
	**100**	184.040	9.400	184.040	19.294	184.040	57.911	184.040	101.249	179.819	9.194	179.819	18.887	179.819	56.635	179.819	98.818
	**200**	86.880	4.680	86.880	9.099	86.880	27.661	86.880	49.433	84.903	4.576	84.903	8.906	84.903	27.030	84.903	48.404
	**300**	56.163	2.971	56.163	6.245	56.163	18.649	56.163	31.968	54.920	2.906	54.920	6.111	54.920	18.236	54.920	31.269
	**500**	31.904	1.654	31.904	3.658	31.904	10.377	31.904	19.371	31.166	1.616	31.166	3.579	31.166	10.131	31.166	18.957
**0.90**	**50**	253.654	12.477	253.654	28.907	253.654	81.873	253.654	145.531	247.388	12.197	247.388	28.274	247.388	79.908	247.388	142.295
	**100**	115.804	5.772	115.804	13.703	115.804	39.790	115.804	66.664	113.121	5.645	113.121	13.407	113.121	38.923	113.121	65.244
	**200**	55.223	2.836	55.223	6.331	55.223	19.963	55.223	33.252	53.942	2.772	53.942	6.195	53.942	19.527	53.942	32.504
	**300**	37.497	1.989	37.497	4.159	37.497	13.205	37.497	22.813	36.652	1.945	36.652	4.061	36.652	12.911	36.652	22.321
	**500**	22.447	1.178	22.447	2.691	22.447	7.397	22.447	14.033	21.913	1.150	21.913	2.632	21.913	7.231	21.913	13.725
**0.95**	**50**	137.695	7.001	137.695	13.881	137.695	45.025	137.695	87.762	134.478	6.852	134.478	13.563	134.478	44.011	134.478	85.869
	**100**	66.546	3.504	66.546	6.250	66.546	20.083	66.546	38.171	65.063	3.429	65.063	6.109	65.063	19.613	65.063	37.279
	**200**	31.766	1.630	31.766	3.342	31.766	10.153	31.766	18.840	31.048	1.593	31.048	3.272	31.048	9.936	31.048	18.422
	**300**	19.653	1.019	19.653	2.216	19.653	6.814	19.653	10.992	19.189	0.995	19.189	2.165	19.189	6.675	19.189	10.747
	**500**	11.605	0.608	11.605	1.171	11.605	3.712	11.605	6.638	11.349	0.594	11.349	1.146	11.349	3.627	11.349	6.491

**Table 2:** The *MSEs* of ratio-type estimators with and without *ME* for $S_{U}^{2}=2,S_{V}^{2}=2$.

with *ME*										without *ME*							
ρ_YX_	*n*	*λ* = 0.1		*λ* = 0.2		*λ* = 0.5		*λ* = 0.75		*λ* = 0.1		*λ* = 0.2		*λ* = 0.5		*λ* = 0.75	
		*t*_*r*_	$t_{rt}^{M}$	*t*_*r*_	$t_{rt}^{M}$	*t*_*r*_	$t_{rt}^{M}$	*t*_*r*_	$t_{rt}^{M}$	*t*_*r*_	$t_{rt}^{M}$	*t*_*r*_	$t_{rt}^{M}$	*t*_*r*_	$t_{rt}^{M}$	*t*_*r*_	$t_{rt}^{M}$
**0.80**	**50**	530.261	25.284	530.261	57.211	530.261	156.005	530.261	289.527	479.085	23.119	479.085	52.303	479.085	142.475	479.085	262.578
	**100**	249.057	12.602	249.057	26.894	249.057	85.979	249.057	151.133	225.648	11.480	225.648	24.573	225.648	78.770	225.648	137.739
	**200**	123.143	6.459	123.143	12.579	123.143	42.432	123.143	68.619	112.691	5.924	112.691	11.521	112.691	38.737	112.691	62.876
	**300**	69.524	3.763	69.524	8.355	69.524	24.843	69.524	45.898	63.114	3.423	63.114	7.647	63.114	22.706	63.114	41.645
	**500**	45.812	2.350	45.812	4.966	45.812	15.700	45.812	28.024	41.919	2.152	41.919	4.549	41.919	14.388	41.919	25.477
**0.85**	**50**	410.111	20.133	410.111	44.685	410.111	135.055	410.111	248.474	371.589	18.426	371.589	40.618	371.589	122.847	371.589	226.454
	**100**	200.315	10.392	200.315	20.588	200.315	67.217	200.315	118.648	183.356	9.556	183.356	18.768	183.356	61.424	183.356	107.715
	**200**	99.735	5.399	99.735	10.656	99.735	28.886	99.735	55.525	90.874	4.932	90.874	9.752	90.874	26.387	90.874	50.505
	**300**	61.278	3.229	61.278	7.257	61.278	18.982	61.278	33.901	55.606	2.936	55.606	6.651	55.606	17.281	55.606	30.994
	**500**	32.018	1.651	32.018	3.986	32.018	12.006	32.018	21.931	29.191	1.507	29.191	3.638	29.191	10.924	29.191	20.086
**0.90**	**50**	256.489	12.043	256.489	29.480	256.489	85.714	256.489	159.826	231.451	10.983	231.451	27.020	231.451	78.634	231.451	145.860
	**100**	131.558	6.913	131.558	13.885	131.558	46.978	131.558	79.715	119.514	6.310	119.514	12.631	119.514	42.646	119.514	72.596
	**200**	62.290	3.115	62.290	7.196	62.290	20.941	62.290	39.433	57.029	2.857	57.029	6.612	57.029	19.123	57.029	35.860
	**300**	40.304	2.118	40.304	4.729	40.304	13.794	40.304	25.997	36.737	1.934	36.737	4.344	36.737	12.563	36.737	23.671
	**500**	24.705	1.277	24.705	2.424	24.705	8.269	24.705	14.898	22.571	1.168	22.571	2.194	22.571	7.552	22.571	13.699
**0.95**	**50**	139.471	6.787	139.471	15.190	139.471	46.045	139.471	84.670	126.366	6.209	126.366	13.878	126.366	41.775	126.366	77.432
	**100**	69.431	3.537	69.431	7.111	69.431	22.772	69.431	38.073	63.299	3.238	63.299	6.492	63.299	20.693	63.299	34.620
	**200**	33.112	1.729	33.112	3.671	33.112	10.626	33.112	19.249	30.096	1.574	30.096	3.353	30.096	9.699	30.096	17.515
	**300**	22.766	1.234	22.766	2.401	22.766	6.861	22.766	12.687	20.769	1.128	20.769	2.189	20.769	6.272	20.769	11.549
	**500**	12.533	0.640	12.533	1.390	12.533	4.045	12.533	7.574	11.411	0.583	11.411	1.272	11.411	3.667	11.411	6.920

**Table 3:** The *RE* of ratio-type estimators with and without *ME* for $S_{U}^{2}=1,S_{V}^{2}=1$.

with *ME*										without *ME*							
ρYX	n	λ = 0.1		λ = 0.2		λ = 0.5		λ = 0.75		λ = 0.1		λ = 0.2		λ = 0.5		λ = 0.75	
		tr	$t_{rt}^{M}$	tr	$t_{rt}^{M}$	tr	$t_{rt}^{M}$	tr	$t_{rt}^{M}$	tr	$t_{rt}^{M}$	tr	$t_{rt}^{M}$	tr	$t_{rt}^{M}$	tr	$t_{rt}^{M}$
**0.80**	**50**	1.206	24.722	1.206	11.987	1.206	4.003	1.206	2.192	1.237	25.299	1.237	12.252	1.237	4.097	1.237	2.246
	**100**	1.329	26.564	1.329	11.069	1.329	3.791	1.329	2.074	1.361	27.171	1.361	11.329	1.361	3.876	1.361	2.123
	**200**	1.367	26.581	1.367	13.084	1.367	4.223	1.367	1.994	1.399	27.181	1.399	13.380	1.399	4.322	1.399	2.041
	**300**	1.396	26.605	1.396	13.271	1.396	4.030	1.396	2.311	1.428	27.196	1.428	13.567	1.428	4.121	1.428	2.360
	**500**	1.441	27.173	1.441	12.759	1.441	4.263	1.441	2.150	1.474	27.777	1.474	13.065	1.474	4.356	1.474	2.198
**0.85**	**50**	1.622	32.775	1.622	16.440	1.622	5.484	1.622	2.916	1.663	33.521	1.663	16.827	1.663	5.612	1.663	2.986
	**100**	1.734	33.953	1.734	16.577	1.734	5.114	1.734	3.202	1.775	34.713	1.775	16.935	1.775	5.230	1.775	3.281
	**200**	1.745	32.393	1.745	17.840	1.745	5.493	1.745	3.244	1.786	33.127	1.786	18.227	1.786	5.621	1.786	3.313
	**300**	1.690	31.953	1.690	14.952	1.690	5.248	1.690	2.856	1.728	32.668	1.728	15.280	1.728	5.367	1.728	2.920
	**500**	1.730	33.371	1.730	16.048	1.730	5.306	1.730	2.932	1.771	34.153	1.771	16.400	1.771	5.435	1.771	2.996
**0.90**	**50**	2.508	50.992	2.508	21.583	2.508	6.673	2.508	4.430	2.572	52.162	2.572	22.066	2.572	6.837	2.572	4.531
	**100**	2.625	52.667	2.625	23.654	2.625	8.079	2.625	4.737	2.687	53.851	2.687	24.178	2.687	8.259	2.687	4.840
	**200**	2.698	52.532	2.698	23.071	2.698	7.934	2.698	4.301	2.762	53.750	2.762	23.578	2.762	8.111	2.762	4.400
	**300**	2.534	47.777	2.534	23.642	2.534	8.120	2.534	4.238	2.593	48.861	2.593	24.210	2.593	8.305	2.593	4.332
	**500**	2.322	44.242	2.322	22.863	2.322	7.033	2.322	4.263	2.379	45.313	2.379	23.374	2.379	7.195	2.379	4.358
**0.95**	**50**	5.017	98.667	5.017	42.849	5.017	12.949	5.017	7.808	5.137	100.809	5.137	43.852	5.137	13.248	5.137	7.981
	**100**	5.016	95.263	5.016	47.359	5.016	13.893	5.016	8.469	5.130	97.338	5.130	48.456	5.130	14.225	5.130	8.672
	**200**	4.678	91.192	4.678	45.902	4.678	13.770	4.678	8.352	4.786	93.256	4.786	46.887	4.786	14.070	4.786	8.541
	**300**	4.517	87.116	4.517	43.333	4.517	15.585	4.517	8.410	4.627	89.195	4.627	44.349	4.627	15.910	4.627	8.602
	**500**	4.909	93.765	4.909	46.023	4.909	14.723	4.909	8.437	5.020	95.867	5.020	47.034	5.020	15.066	5.020	8.628

**Table 4:** The *RE* of ratio-type estimators with and without *ME* for $S_{U}^{2}=2,S_{V}^{2}=2$.

with *ME*										without *ME*							
ρ_YX_	*n*	*λ* = 0.1		*λ* = 0.2		*λ* = 0.5		*λ* = 0.75		*λ* = 0.1		*λ* = 0.2		*λ* = 0.5		*λ* = 0.75	
		*t*_*r*_	$t_{rt}^{M}$	*t*_*r*_	$t_{rt}^{M}$	*t*_*r*_	$t_{rt}^{M}$	*t*_*r*_	$t_{rt}^{M}$	*t*_*r*_	$t_{rt}^{M}$	*t*_*r*_	$t_{rt}^{M}$	*t*_*r*_	$t_{rt}^{M}$	*t*_*r*_	$t_{rt}^{M}$
**0.80**	**50**	1.158	24.280	1.158	11.309	1.158	3.449	1.158	2.099	1.281	26.555	1.281	12.370	1.281	3.776	1.281	2.315
	**100**	1.330	26.288	1.330	11.351	1.330	3.694	1.330	2.055	1.468	28.858	1.468	12.423	1.468	4.032	1.468	2.255
	**200**	1.226	23.382	1.226	11.513	1.226	3.604	1.226	2.228	1.340	25.493	1.340	12.570	1.340	3.947	1.340	2.431
	**300**	1.314	24.269	1.314	11.792	1.314	3.856	1.314	2.037	1.447	26.683	1.447	12.884	1.447	4.219	1.447	2.245
	**500**	1.328	25.884	1.328	11.605	1.328	3.848	1.328	2.067	1.451	28.263	1.451	12.669	1.451	4.199	1.451	2.274
**0.85**	**50**	1.686	34.353	1.686	13.845	1.686	4.858	1.686	2.690	1.861	37.537	1.861	15.231	1.861	5.340	1.861	2.951
	**100**	1.723	33.208	1.723	14.454	1.723	4.731	1.723	2.435	1.882	36.114	1.882	15.855	1.882	5.177	1.882	2.682
	**200**	1.507	27.834	1.507	14.005	1.507	5.076	1.507	2.855	1.654	30.471	1.654	15.302	1.654	5.557	1.654	3.138
	**300**	1.565	29.690	1.565	14.479	1.565	4.941	1.565	2.867	1.724	32.652	1.724	15.797	1.724	5.428	1.724	3.136
	**500**	1.691	32.795	1.691	15.659	1.691	4.737	1.691	2.776	1.855	35.921	1.855	17.156	1.855	5.206	1.855	3.031
**0.90**	**50**	2.215	47.182	2.215	20.797	2.215	7.127	2.215	3.939	2.455	51.733	2.455	22.690	2.455	7.769	2.455	4.316
	**100**	2.193	41.726	2.193	22.957	2.193	6.762	2.193	3.980	2.414	45.713	2.414	25.236	2.414	7.448	2.414	4.370
	**200**	2.525	50.492	2.525	22.053	2.525	6.873	2.525	3.879	2.758	55.053	2.758	24.000	2.758	7.527	2.758	4.266
	**300**	2.576	49.017	2.576	22.798	2.576	7.271	2.576	3.672	2.826	53.689	2.826	24.819	2.826	7.983	2.826	4.033
	**500**	2.352	45.500	2.352	22.251	2.352	6.548	2.352	4.342	2.574	49.757	2.574	24.582	2.574	7.170	2.574	4.722
**0.95**	**50**	4.646	95.476	4.646	40.308	4.646	13.616	4.646	7.490	5.128	104.354	5.128	44.119	5.128	15.008	5.128	8.190
	**100**	4.745	93.148	4.745	39.440	4.745	13.697	4.745	7.905	5.205	101.749	5.205	43.201	5.205	15.073	5.205	8.694
	**200**	4.342	83.183	4.342	40.200	4.342	15.303	4.342	8.318	4.778	91.370	4.778	44.015	4.778	16.765	4.778	9.142
	**300**	4.615	85.144	4.615	41.878	4.615	14.697	4.615	7.369	5.058	93.165	5.058	45.927	5.058	16.075	5.058	8.094
	**500**	4.383	85.802	4.383	39.401	4.383	14.347	4.383	7.523	4.814	94.210	4.814	43.074	4.814	15.823	4.814	8.234

From the numerical findings summarized in Tables 1 and 2, it is observed that the *MSEs* of the memory-type ratio estimator are less than the *MSE*s of the conventional ratio estimator in the presence of *ME*. Equivalently, the *RE*s presented in Tables 3 and 4 shown that the proposed ratio estimator is more efficient than the conventional ratio estimator. The effect of *ρ*_*YX*_ is observed as it increases up to 1, the *MSE* decreases and the efficiency of the proposed memory-type ratio estimator increases which revealed that the use of the auxiliary variable, correlated with the survey variable may enhance the efficiency of the proposed estimator. The role of sample size is significant in the efficiency of the proposed estimator. The *MSEs* of the proposed estimator decrease as the sample size increases. Similarly, the weight *λ* assigned to the current and previous values has the great impact on the efficiency of the proposed memory-type ratio estimator as shown in Tables 3 and 4. It is further noticed that, when the value of *λ* goes down, the larger weight is assigned to previous values and hence increases the efficiency of the proposed estimators. For *λ* = 1, the efficiency of proposed estimators based on *EWMA* statistic will be as good as conventional estimators.

To check the efficiency of the memory-type product estimator, we generated a bivariate population of size 1000 having negative correlation between the concerned variable and the auxiliary variable. The detail of the population parameter is

$$\mu_{y}=\mu_{x}=5,\;\mu_{U}=\mu_{V}=0,\;S_{y}^{2}=5,\;S_{x}^{2}=S_{U}^{2}=S_{V}^{2}=2\;\mathrm{and}\;\rho_{yx}=-0.9432$$

The results of *MSE* and *RE* of proposed and conventional ratio and product estimators are summarized in Tables 5 and 6 displayed that the product type estimators perform better than the ratio estimators in the presence of *ME* for the population having a negatively correlated data with the study variable. Moreover, the proposed memory-type product estimator found to be more efficient than the conventional product estimator on various sample sizes and *λ*. The *MSE*s of the product eestimator decreases as the sample size increases from 50 to 500.

**Table 5:** The *MSE* of proposed and conventional ratio and product estimators.

*Estimator*	*n*	With *ME*				Without *ME*			
		*λ* = 0.1	*λ* = 0.2	*λ* = 0.5	*λ* = 0.75	*λ* = 0.1	*λ* = 0.2	*λ* = 0.5	*λ* = 0.75
***t***_***r***_	50	4.4592	4.4592	4.4592	4.4592	3.8368	3.8368	3.8368	3.8368
	100	1.8961	1.8961	1.8961	1.8961	0.6383	0.6383	0.6383	0.6383
	200	1.0319	1.0319	1.0319	1.0319	0.4856	0.4856	0.4856	0.4856
	300	0.6234	0.6234	0.6234	0.6234	0.2753	0.2753	0.2753	0.2753
	500	0.3668	0.3668	0.3668	0.3668	0.1490	0.1490	0.1490	0.1490
$t_{rt}^{M}$	50	0.2208	0.4474	1.2986	2.4348	0.0548	0.1737	0.5324	0.8282
	100	0.0960	0.2000	0.5683	1.1030	0.0244	0.0673	0.1544	0.3395
	200	0.0546	0.1000	0.3293	0.5392	0.0222	0.0269	0.1239	0.1959
	300	0.0344	0.0725	0.2092	0.3555	0.0131	0.0301	0.0674	0.1270
	500	0.0215	0.0419	0.1163	0.1879	0.0076	0.0156	0.0481	0.0607
***t***_***p***_	50	0.3590	0.3590	0.3590	0.3590	0.2410	0.2410	0.2410	0.2410
	100	0.2434	0.2434	0.2434	0.2434	0.1129	0.1129	0.1129	0.1129
	200	0.1459	0.1459	0.1459	0.1459	0.0554	0.0554	0.0554	0.0554
	300	0.0941	0.0941	0.0941	0.0941	0.0342	0.0342	0.0342	0.0342
	500	0.0619	0.0619	0.0619	0.0619	0.0207	0.0207	0.0207	0.0207
$t_{pt}^{M}$	50	0.0254	0.0407	0.1102	0.2587	0.0124	0.0246	0.0816	0.1383
	100	0.0144	0.0315	0.0892	0.1635	0.0062	0.0123	0.0352	0.0668
	200	0.0077	0.0198	0.0511	0.0894	0.0034	0.0062	0.0187	0.0348
	300	0.0050	0.0115	0.0432	0.0636	0.0022	0.0041	0.0124	0.0207
	500	0.0033	0.0071	0.0197	0.0407	0.0015	0.0025	0.0069	0.0119

**Table 6:** The *RE* of proposed and conventional ratio and product estimators.

*Estimator*	*n*	With *ME*				Without *ME*			
		*λ* = 0.1	*λ* = 0.2	*λ* = 0.5	*λ* = 0.75	*λ* = 0.1	*λ* = 0.2	*λ* = 0.5	*λ* = 0.75
***t***_***r***_	50	0.2353	0.2353	0.2353	0.2353	0.1872	0.1872	0.1872	0.1872
	100	0.2541	0.2541	0.2541	0.2541	0.5830	0.5830	0.5830	0.5830
	200	0.2569	0.2569	0.2569	0.2569	0.5400	0.5400	0.5400	0.5400
	300	0.2612	0.2612	0.2612	0.2612	0.5770	0.5770	0.5770	0.5770
	500	0.2651	0.2651	0.2651	0.2651	0.6526	0.6526	0.6526	0.6526
$t_{rt}^{M}$	50	4.7511	2.3592	0.7644	0.4174	13.1064	4.4738	1.2966	0.8598
	100	5.0208	2.2985	0.7883	0.4258	15.2642	6.1277	2.2826	1.1285
	200	4.8592	2.3805	0.7787	0.4434	11.8074	7.7428	1.9419	1.1181
	300	4.7378	2.3484	0.7972	0.4366	12.1122	5.5821	2.4404	1.1821
	500	4.5274	2.3416	0.8053	0.4462	12.8730	6.2903	1.9403	1.3698
***t***_***p***_	50	2.0009	2.0009	2.0009	2.0009	4.3535	4.3535	4.3535	4.3535
	100	1.5291	1.5291	1.5291	1.5291	4.2698	4.2698	4.2698	4.2698
	200	1.7969	1.7969	1.7969	1.7969	4.7820	4.7820	4.7820	4.7820
	300	1.6882	1.6882	1.6882	1.6882	4.7571	4.7571	4.7571	4.7571
	500	1.5720	1.5720	1.5720	1.5720	4.7040	4.7040	4.7040	4.7040
$t_{pt}^{M}$	50	28.3010	19.0937	6.2627	2.7526	84.7392	42.9018	12.1584	7.3471
	100	25.7807	13.1008	3.9521	2.3443	77.5606	37.4096	12.7207	7.0364
	200	34.1647	10.5142	4.7110	2.4508	77.6755	38.3420	13.6985	6.8772
	300	31.7891	14.6844	3.8049	2.3582	74.7489	41.3211	13.5058	7.5054
	500	29.4871	13.7350	4.7378	2.0414	63.2460	39.2792	13.6075	7.0393

## 6. Real-data application

A real data is taken from [30] to confirm the application of memory-type ratio estimator proposed in this paper. The data set contains the true consumption expenditure as study variable *Y* and true income as an auxiliary variable *X* whereas the variables contaminated with *ME* are taken *y* as measured consumption expenditure and *x* as measured income with the respective population parameters are *N* = 10, *μ*_*y*_ = 127, *μ*_*x*_ = 170, $S_{y}^{2}=1420,$ $S_{x}^{2}=3666.67,$ $S_{U}^{2}=36,$ $S_{V}^{2}=41.24,$ and *ρ*_*yx*_ = 0.964.

By considering regular time intervals, twenty samples, each of size 4, with *λ* = 0.2 are chosen by using *SRSWOR*. Here, we only considered the conventional and proposed memory-type ratio estimators as the correlation coefficient between the study and the auxiliary variables is positive. The results of all the estimators (except the product-type estimators) in the presence and absence of *ME* are computed and reported in Table 7. It is clear from the numerical findings given in Table 8, that the proposed memory-type ratio estimator under *ME* is more efficient than the conventional ratio estimator in terms of *MSE* and *RE*.

**Table 7:** EWMA statistics and ratio estimators.

Without *ME*				With *ME*			
${\hat{V}}_{t}$	${\hat{W}}_{t}$	*t*_*r*_	${\hat{t}}_{rt}^{M}$	${\hat{V}}_{t}$	${\hat{W}}_{t}$	*t*_*r*_	${\hat{t}}_{rt}^{M}$
198.6156	599.2181	1212.0690	1215.3461	205.8157	607.4667	1239.1057	1242.3027
582.7457	1451.1258	1599.3065	1472.4665	595.7057	1465.9733	1612.7841	1489.9687
862.8768	2352.6521	1220.4678	1344.8149	880.4449	2372.7787	1234.0786	1360.5558
1085.3720	3073.8730	1215.5140	1294.6850	1106.6260	3098.2230	1229.1590	1309.6630
1373.7060	3784.1830	1398.5250	1331.0460	1397.9090	3811.9120	1409.6730	1344.6450
1337.9150	3892.4310	1012.7920	1260.3150	1364.4780	3922.8630	1033.4560	1275.3660
1169.9513	3372.3631	1413.4889	1272.0521	1198.4018	3404.9568	1468.7670	1290.5126
1039.8711	3189.6418	774.7890	1195.3884	1069.8315	3223.9654	814.8075	1216.7362
1061.5790	3096.7980	1545.0200	1256.9290	1092.7470	3132.5060	1569.7000	1279.0850
1072.7030	3269.1900	1034.7690	1203.1250	1104.8380	3306.0050	1057.1000	1225.3680
1258.4810	3700.4370	1352.7370	1246.9960	1291.3890	3738.1370	1366.6780	1266.6980
1578.2010	4285.4340	1581.1760	1350.3270	1611.7270	4323.8430	1591.1940	1366.7620
1523.5610	4113.4320	1396.9120	1358.0850	1557.5820	4152.4080	1418.3700	1375.3790
1586.2890	4209.1640	1466.9580	1381.8410	1620.7060	4248.5930	1482.3890	1398.7190
1464.1323	3732.4156	1959.4623	1438.3406	1498.8660	3772.2080	1986.8840	1456.9300
1563.9600	4097.6840	1295.0120	1399.4540	1598.9470	4137.7660	1309.0460	1416.9010
1456.4940	3963.2320	1098.9350	1347.5060	1491.6840	4003.5460	1123.9380	1366.1660
1344.9232	3935.6702	861.3453	1252.9976	1380.2749	3976.1703	886.2960	1272.8348
1247.7569	3373.6209	2798.9482	1356.1419	1283.2383	3414.2696	2813.1455	1378.1006
1077.2657	3063.9814	794.0275	1289.1639	1112.8508	3104.7490	847.1986	1314.2618

**Table 8:** The *MSE* and *RE* of *EWMA* statistic and ratio estimators for real population.

*Estimators*	*With ME*		*Without ME*	
	*MSE*	*RE*	*MSE*	*RE*
*t*_*r*_	*203080*.*4000*	*2*.*4039*	*199117*.*9000*	*2*.*4517*
${\hat{t}}_{rt}^{M}$	*5950*.*7630*	*82*.*0364*	*5715*.*0350*	*85*.*4201*

## 7. Conclusion

In this paper, we have suggested memory-type ratio and product estimators based on *EWMA* statistics for estimating the population variance in the presence and absence of *ME*. The proposed estimators use the current as well as previous sample information. The reported results show that the proposed estimators estimated the population variance more efficiently than the conventional estimators. The ratio (memory-type and conventional) estimators perform well for the populations having a positive correlation between the study and the auxiliary variables, whereas the product (memory-type and conventional) estimators perform better for negatively correlated populations. Further, the *REs* of the proposed estimators increase, and the *MSE*s decrease as the sample size increases. The results of real data application confirm the findings obtained from the simulation. Consequently, based on numerical results, it is suggested that the proposed memory-type ratio and product estimators based on *EWMA* statistics are superior and more useful for estimating population variance for the time-scaled surveys.

Current manuscript uses only one auxiliary variable to estimate the population variance. This approach can be extended to estimate various population characteristics using two or more auxiliary variables in the presence of *ME* and non-response.

## Supporting information

S1 Appendix (DOCX)

## References

[1] Kadilar C, Cingi H. Ratio estimators for the population variance in simple and stratified random sampling. Applied Mathematics and Computation. 2006;173(2):1047–1059. 10.1016/j.amc.2005.04.032.

[2] Shabbir J, Gupta S A T. On Improvement in Variance Estimation. Communications in Statistics - Theory and Methods. 2007;36(12):2177–2185. 10.1080/03610920701215092.

[3] Yadav S K, Kadilar C. Improved exponential type ratio estimator of the population variance. Revista Colombiana de Estadistica. 2013;36(1):145–152.

[4] Adichwal N K, Sharma P, Singh R. Generalized Class of Estimators for Population Variance Using Information on Two Auxiliary Variables. International Journal of Applied and Computational Mathematics. 2017; 3(2):651–661. 10.1007/s40819-015-0119-6.

[5] Singh H P, Pal S K, Yadav A. A study on the chain ratio-ratio-type exponential estimator for finite population variance. Communications in Statistics - Theory and Methods. 2017;47(6):1442–1458. 10.1080/03610926.2017.1321124.

[6] Ismail M, Kanwal N, Shahbaz M Q. Generalized ratio-product-type estimator for variance using auxiliary information in simple random sampling. Kuwait Journal of Science. 2018;45(1):79–88.

[7] Bhat M A. An Improvement in Variance Estimator for the Estimation of Population Variance, Using Known Values of Auxiliary Information. International Journal of Pure & Applied Bioscience. 2018; 6(5):135–138. 10.18782/2320-7051.6842.

[8] Javed K, Jamal N, Hanif M, Ali M, Shahzad U, Victoria Garcia Luengo A. Improved Estimator of Finite Population Variance Using Coefficient of Quartile Deviation. Asian Journal of Advanced Research and Reports. 2018;1(3):1–6. 10.9734/ajarr/2018/v1i313065.

[9] Yadav S K, Sharma D K, Mishra S S. Searching efficient estimator of population variance using tri-mean and third quartile of auxiliary variable. International Journal of Business and Data Analytics. 2019;1(1): 30. 10.1504/ijbda.2019.098830.

[10] Qureshi M N, Riaz N Hanif M. Generalized estimator for the estimation of rare and clustered population variance in adaptive cluster sampling. Journal of Statistical Computation and Simulation. 2019;89(11):2084–2101.

[11] Singh G N, Bhattacharyya D, Bandyopadhyay A. Calibration estimation of population variance under stratified successive sampling in presence of random non response. Communications in Statistics - Theory and Methods. 2020;0(0):1–23. 10.1080/03610926.2020.1719158.

[12] Hansen M H, William N, Hurwitz E, Marks W P M. Response Errors in Survey. JASA. 1951; 46: 147 – 190.

[13] Singh H P, Karpe N. A class of estimators using auxiliary information for estimating finite population variance in presence of measurement errors. Communications in Statistics - Theory and Methods. 2009a;38(5):734–741. 10.1080/03610920802290713.

[14] Misra S, Yadav D K, ika D, Kr Shukla A. On Estimation of Population Coefficient of Variation in Presence of Measurement Errors. International Journal of Mathematics Trends and Technology. 2017;51(4):307–311. 10.14445/22315373/ijmtt-v51p540.

[15] Khalil S, Noor-ul-Amin M, Hanif M. Estimation of population mean for a sensitive variable in the presence of measurement error. Journal of Statistics and Management Systems. 2018;21(1):81–91. 10.1080/09720510.2017.1367478.

[16] Zahid E, Shabbir J. Estimation of finite population mean for a sensitive variable using dual auxiliary information in the presence of measurement errors. PLoS ONE. 2019;14(2):1–17. doi: 10.1371/journal.pone.0212111 30742674 PMC6370250

[17] Khalil S, Gupta S, Hanif M. Estimation of finite population mean in stratified sampling using scrambled responses in the presence of measurement errors. Communications in Statistics Theory and Methods. 2019;48(6):1553–1561. doi: 10.1080/03610926.2018.1435817

[18] Singh R, Bouza C, Mishra M. Estimation in stratified random sampling in the presence of errors. Revista Investigacion Operacional. 2020; 41(1):123–135.

[19] Singh H P, Karpe N. Estimation of Population Variance Using Auxiliary Information in the Presence of Measurement Errors. Statistics in Transition. 2008;9(3):443–470.

[20] Diana G, Giordan M. Finite population variance estimation in presence of measurement errors. Communications in Statistics - Theory and Methods. 2012;41(23):4302–4314. 10.1080/03610926.2011.573165.

[21] Sharma P, Singh R. A generalized class of estimators for finite population variance in presence of measurement errors. Journal of Modern Applied Statistical Methods. 2013;12(2):231–241. 10.22237/jmasm/1383279120.

[22] Singh H P, Pal S K. Improved estimation of finite population variance using auxiliary information in presence of measurement errors. Investigacion Operacional. 2016;37(2):147–162.

[23] Misra S, Kumari D, Yadav D K. Some Improved Estimators for Estimating Population Variance in the Presence of Measurement Errors. Journal of Statistics Applications & Probability. 2016;5(2):311–320. 10.18576/jsap/050212.

[24] Masood S, Shabbir J. Generalized multi-phase regression-type estimators under the effect of measuemnent error to estimate the population variance. Hacettepe Journal of Mathematics and Statistics. 2016;45(4):1297–1306. 10.15672/HJMS.201612116352.

[25] Roberts S W. Control Chart Tests Based on Geometric Moving Averages. Technometrics 1. 1959;3(2):39–50.

[26] Noor-ul-amin M. Memory type estimators of population mean using exponentially weighted moving averages for time scaled surveys. Communications in Statistics - Theory and Methods. 2019;47(24):1–12. 10.1080/03610926.2019.1670850.

[27] Aslam I, Noor-ul-Amin M, Yasmeen U, Hanif M. Memory type ratio and product estimators in stratified sampling. Journal of Reliability and Statistical Studies. 2020; 13(1):1–15.

[28] Singh H P, Karpe N. A General Procedure for Estimating the General Parameter Using Auxiliary Information in Presence of Measurement Errors. Communications for Statistical Applications and Methods. 2009b;16(5):821–840. 10.5351/ckss.2009.16.5.821.

[29] Isaki C T. Variance estimation using auxiliary information. Journal of the American Statistical Association. 1983; 78(381):117–123. 10.1080/01621459.1983.10477939.

[30] Gujarati D N. Basic Econometrics (Fourth Edi). The McGraw−Hill Companies. 2004.

