# Correction: Memory-type variance estimators using exponentially weighted moving average statistic in presence of measurement error for time-scaled surveys

**DOI:** 10.1371/journal.pone.0310870

**Published:** 2024-09-17

**Authors:** Muhammad Nouman Qureshi, Osama Abdulaziz Alamri, Naureen Riaz, Ayesha Iftikhar, Muhammad Umair Tariq, Muhammad Hanif

An additional affiliation is missing for the first author. Muhammad Nouman Qureshi is also affiliated with National College of Business Administration and Economics in Lahore, Pakistan.

After this article [1] was published, the authors also made the following clarifications:

- For the real data for the true consumption expenditure study, the dataset found on page 485, Chapter 13, Example 13.2 of the book Basic Econometrics, 4th Edition, by Gujarati [2] was used.
- Multiple Indicator Cluster Surveys (MICS) is mentioned in the first paragraph of the Introduction in [1] as it provides context and rationale for the study. However, for the empirical analysis presented in [1], the MICS data was not directly utilized.

## References

[1] Qureshi MN, Alamri OA, Riaz N, Iftikhar A, Tariq MU, Hanif M (2023) Memory-type variance estimators using exponentially weighted moving average statistic in presence of measurement error for time-scaled surveys. PLoS ONE 18(11): e0277697. 10.1371/journal.pone.0277697 37944483 PMC10635740

[2] Gujarati D N. Basic Econometrics (Fourth Edi). The McGraw−Hill Companies. 2004

