# Supplementary material for: Memory-type variance estimators using exponentially weighted moving average statistic in presence of measurement error for time-scaled surveys
Source: PLoS One. 2023 Nov 9;18(11):e0277697. doi: 10.1371/journal.pone.0277697 (PMC10635740; doi:10.1371/journal.pone.0277697)
Supplement: S1 Appendix — (DOCX) [file pone.0277697.s001.docx]

**Appendix A:**

**A.1**

$$\xi_{o}=\frac{\hat{s}_{y}^{2}-S_{y}^{2}}{S_{y}^{2}}.$$

Or,

$$\xi_{o}=\frac{s_{y}^{2}-S_{U}^{2}-S_{y}^{2}}{S_{y}^{2}}.$$

Or,

$$\xi_{o}=\frac{1}{S_{y}^{2}}\left[ s_{u}^{2}+s_{y}^{2}+2s_{uy}^{2}-S_{U}^{2}-S_{y}^{2} \right].$$

Or,

$$\xi_{o}=\frac{1}{S_{y}^{2}}\left[ (s_{u}^{2}-S_{U}^{2})+(s_{y}^{2}-S_{y}^{2})+2s_{uy}^{2} \right].$$

Square and applying expectation on both sides of above equation up to first degree, we have

$$E\left( \xi_{o} \right)^{2}=\frac{1}{S_{y}^{4}}\left[ E{(s_{u}^{2}-S_{U}^{2})}^{2}+E{(s_{y}^{2}-S_{y}^{2})}^{2}+4n{(n-1)}^{-2}E{(s_{u}-S_{U})}^{2}{(s_{y}-S_{y})}^{2} \right].$$

$$E\left( \xi_{o} \right)^{2}=\frac{1}{S_{y}^{4}}\left[ \frac{S_{y}^{4}}{n}(\beta_{2y}-3)+\frac{S_{U}^{4}}{n}(\beta_{2u}-3)+4\frac{S_{U}^{2}S_{y}^{2}}{n} \right].$$

$$=\frac{1}{n}\left[ \gamma_{2y}+\gamma_{2u}\frac{S_{u}^{4}}{S_{y}^{4}}+2{(1+\frac{S_{u}^{2}}{S_{y}^{2}})}^{2} \right].$$

$$=\frac{A_{y}}{n}.$$

**A.2**

$$\xi_{1}=\frac{\hat{s}_{x}^{2}-S_{x}^{2}}{S_{x}^{2}}.$$

Or,

$$\xi_{1}=\frac{s_{x}^{2}-S_{V}^{2}-S_{x}^{2}}{S_{x}^{2}}.$$

Or,

$$\xi_{1}=\frac{1}{S_{x}^{2}}\left[ s_{v}^{2}+s_{x}^{2}+2s_{vx}^{2}-S_{V}^{2}-S_{x}^{2} \right].$$

Or,

$$\xi_{1}=\frac{1}{S_{x}^{2}}\left[ (s_{v}^{2}-S_{V}^{2})+(s_{x}^{2}-S_{x}^{2})+2s_{vx}^{2} \right].$$

Squaring and applying expectation on both sides of above equation up to first degree, we have

$$E\left( \xi_{1} \right)^{2}=\frac{1}{S_{x}^{4}}\left[ E{(s_{v}^{2}-S_{V}^{2})}^{2}+E{(s_{x}^{2}-S_{x}^{2})}^{2}+4n{(n-1)}^{-2}E{(s_{v}-S_{V})}^{2}{(s_{x}-S_{x})}^{2} \right].$$

$$E\left( \xi_{1} \right)^{2}=\frac{1}{S_{x}^{4}}\left[ \frac{S_{x}^{4}}{n}(\beta_{2x}-3)+\frac{S_{V}^{4}}{n}(\beta_{2v}-3)+4\frac{S_{V}^{2}S_{x}^{2}}{n} \right].$$

$$=\frac{1}{n}\left[ \gamma_{2x}+\gamma_{2v}\frac{S_{v}^{4}}{S_{x}^{4}}+2{(1+\frac{S_{v}^{2}}{S_{x}^{2}})}^{2} \right].$$

$$E\left( \xi_{1} \right)^{2}=\frac{A_{x}}{n}.$$

**A.3**

$$\xi_{o}\xi_{1}=\left( \frac{\hat{s}_{y}^{2}-S_{y}^{2}}{S_{y}^{2}} \right)\left( \frac{\hat{s}_{x}^{2}-S_{x}^{2}}{S_{x}^{2}} \right).$$

Or,

$$\xi_{o}\xi_{1}=\left( \frac{s_{y}^{2}-S_{U}^{2}-S_{y}^{2}}{S_{y}^{2}} \right)\left( \frac{s_{x}^{2}-S_{V}^{2}-S_{x}^{2}}{S_{x}^{2}} \right).$$

Or,

$$\xi_{o}\xi_{1}=\frac{1}{S_{y}^{2}S_{x}^{2}}\left[ s_{u}^{2}+s_{y}^{2}+2s_{uy}^{2}-S_{U}^{2}-S_{y}^{2} \right]\left[ s_{v}^{2}+s_{x}^{2}+2s_{vx}^{2}-S_{V}^{2}-S_{x}^{2} \right].$$

Or,

$$\xi_{o}\xi_{1}=\frac{1}{S_{y}^{2}S_{x}^{2}}\left[ (s_{u}^{2}-S_{U}^{2})+(s_{y}^{2}-S_{y}^{2})+2s_{uy}^{2} \right]\left[ (s_{v}^{2}-S_{V}^{2})+(s_{x}^{2}-S_{x}^{2})+2s_{vx}^{2} \right].$$

Applying expectation on both sides of above equation up to first degree of approximation, we have

$$E(\xi_{o}\xi_{1})=\frac{1}{S_{y}^{2}S_{x}^{4}}E\left[ (s_{u}^{2}-S_{U}^{2})+(s_{y}^{2}-S_{y}^{2})+2s_{uy}^{2} \right]\left[ (s_{v}^{2}-S_{V}^{2})+(s_{x}^{2}-S_{x}^{2})+2s_{vx}^{2} \right].$$

Since $COV\left( X,Y \right)\neq0.\mathrm{and} COV\left( X,U \right)=COV\left( X,V \right)=COV\left( U,Y \right)=COV\left( V,Y \right)=COV\left( U,V \right)=0.$

We have,

$$E(\xi_{o}\xi_{1})=\frac{1}{n}\left[ \frac{\mu_{22}(X,Y)-S_{x}^{2}S_{y}^{2}}{S_{x}^{2}S_{y}^{2}} \right].$$

$$E\left( \xi_{o}\xi_{1} \right)=\frac{1}{n}\left[ \frac{\mu_{22}(X,Y)}{S_{x}^{2}S_{y}^{2}}-1 \right]$$

$$E\left( \xi_{o}\xi_{1} \right)=\frac{\delta-1}{n}.$$

Mathematical derivation of memory type ratio and product estimators is given as:

We may re-write the memory-type ratio estimator in term of sampling errors as

$$\hat{t}_{rt}^{M}=\hat{V}_{t}\left( \frac{S_{x}^{2}}{\hat{W}_{t}} \right)= S_{y}^{2}\left( 1+\xi_{0t} \right)\left( \frac{S_{x}^{2}}{S_{x}^{2}\left( 1+\xi_{1t} \right)} \right).$$

$$\hat{t}_{rt}^{M}=S_{y}^{2}\left( 1+\xi_{0t} \right)\left( 1+\xi_{1t} \right)^{-1}$$

Simplifying and applying Taylor series up to the second order on above equation, we have

$$\hat{t}_{rt}^{M}\approx S_{y}^{2}\left( 1+\xi_{0t} \right)\left( 1-\xi_{1t}+{\xi_{1t}}^{2} \right).$$

$$\left( \hat{t}_{rt}^{M}-S_{y}^{2} \right)\approx S_{y}^{2}\left( \xi_{0t}-\xi_{1t}-\xi_{0t}\xi_{1t}+{\xi_{1t}}^{2} \right).$$

Squaring and retaining the terms of the second order of the above equation, we get

$$\left( \hat{t}_{rt}^{M}-S_{y}^{2} \right)^{2}\approx S_{y}^{4}\left( \xi_{0t}^{2}+\xi_{1t}^{2}-\xi_{0t}\xi_{1t} \right).$$

Applying expectation on both sides, we have the MSE of $\hat{t}_{rt}^{M}$ as

$${E\left( \hat{t}_{rt}^{M}-S_{y}^{2} \right)}^{2}\approx S_{y}^{4}E\left( \xi_{0t}^{2}+\xi_{1t}^{2}-\xi_{0t}\xi_{1t} \right).$$

Or,

$$MSE\left( \hat{t}_{rt}^{M} \right)\approx\left( \frac{\lambda}{n\left( 2-\lambda\right)} \right)S_{y}^{4}\left[ A_{y}+A_{x}-2\left( \delta-1 \right) \right].$$

Similarly, the *MSE* of memory-type product estimator is

$$MSE\left( \hat{t}_{pt}^{M} \right)\approx\left( \frac{\lambda}{n\left( 2-\lambda\right)} \right)S_{y}^{4}\left[ A_{y}+A_{x}+2\left( \delta-1 \right) \right].$$
